# Hierarchical macro-microporous WPU-ECM scaffolds combined with Microfracture Promote *in Situ* Articular Cartilage Regeneration in Rabbits

**DOI:** 10.1016/j.bioactmat.2020.12.009

**Published:** 2020-12-22

**Authors:** Mingxue Chen, YangYang Li, Shuyun Liu, Zhaoxuan Feng, Hao Wang, Dejin Yang, Weimin Guo, Zhiguo Yuan, Shuang Gao, Yu Zhang, Kangkang Zha, Bo Huang, Fu Wei, Xinyu Sang, Qinyu Tian, Xuan Yang, Xiang sui, Yixin Zhou, Yufeng Zheng, Quanyi Guo

**Affiliations:** a Department of Orthopaedic Surgery, Peking University Fourth School of Clinical Medicine, Beijing Jishuitan Hospital, No. 31 Xinjiekou East Street, Xicheng District, Beijing, 100035, People's Republic of China; b Institute of Orthopedics, Chinese PLA General Hospital, Beijing Key Lab of Regenerative Medicine in Orthopedics, Key Laboratory of Musculoskeletal Trauma & War Injuries PLA, No.28 Fuxing Road, Haidian District, Beijing, 100853, People's Republic of China; c Academy for Advanced Interdisciplinary Studies, Peking University, No. 5 Yiheyuan Road, Haidian District, Beijing, 100871, People's Republic of China; d School of Material Science and Engineering, University of Science and Technology Beijing, No. 30 Xueyuan Road, Haidian District, Beijing, 100083, People's Republic of China; e Department of Bone and Joint Surgery, The Affiliated Hospital of Southwest Medical University, No. 25 Taiping Road, Luzhou, 646000, People's Republic of China; f Department of Materials Science and Engineering, College of Engineering, Peking University, No. 5 Yiheyuan Road, Haidian District, Beijing, 100871, China

**Keywords:** *Extracellular matrix*, *Waterborne polyurethane*, *Low-temperature deposition manufacturing*, *Articular cartilage*, *Tissue engineering*

## Abstract

Tissue engineering provides a promising avenue for treating cartilage defects. However, great challenges remain in the development of structurally and functionally optimized scaffolds for cartilage repair and regeneration. In this study, decellularized cartilage extracellular matrix (ECM) and waterborne polyurethane (WPU) were employed to construct WPU and WPU-ECM scaffolds by water-based 3D printing using low-temperature deposition manufacturing (LDM) system, which combines rapid deposition manufacturing with phase separation techniques. The scaffolds successfully achieved hierarchical macro‐microporous structures. After adding ECM, WPU scaffolds were markedly optimized in terms of porosity, hydrophilia and bioactive components. Moreover, the optimized WPU-ECM scaffolds were found to be more suitable for cell distribution, adhesion, and proliferation than the WPU scaffolds. Most importantly, the WPU-ECM scaffold could facilitate the production of glycosaminoglycan (GAG) and collagen and the upregulation of cartilage-specific genes. These results indicated that the WPU-ECM scaffold with hierarchical macro‐microporous structures could recreate a favorable microenvironment for cell adhesion, proliferation, differentiation, and ECM production. *In vivo* studies further revealed that the hierarchical macro‐microporous WPU-ECM scaffold combined with the microfracture procedure successfully regenerated hyaline cartilage in a rabbit model. Six months after implantation, the repaired cartilage showed a similar histological structure and mechanical performance to that of normal cartilage. In conclusion, the hierarchical macro‐microporous WPU-ECM scaffold may be a promising candidate for cartilage tissue engineering applications in the future.

## Highlights

• Hierarchical macro‐microporous scaffolds could be fabricated by low-temperature deposition manufacturing.
• Waterborne polyurethane (WPU) scaffolds were optimized by adding decellularized cartilage extracellular matrix (ECM).
• The WPU-ECM scaffold provided a suitable microenvironment for cell attachment, proliferation, and differentiation *in vitro.*
• The paradigm of WPU-ECM scaffold and microfracture (MF) has great potential for clinical application in cartilage repair.

## Graphical abstract

**Figure:**
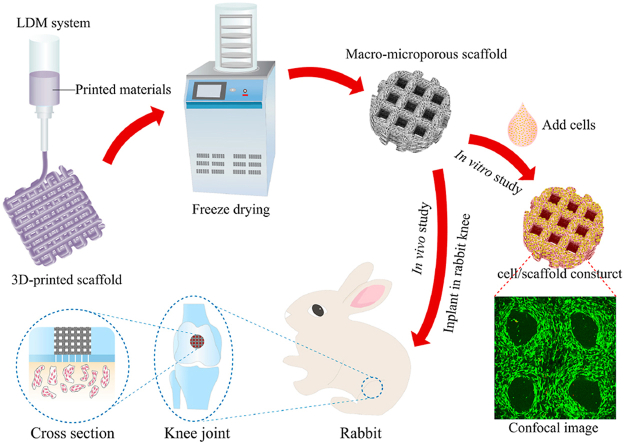

## 1 Introduction

Articular cartilage is an avascular and aneural tissue. Once cartilage defects occur, articular cartilage is difficult to regenerate due to its limited intrinsic self-repair capacity [1]. Hence, the repair of cartilage defects remains a great challenge in clinical treatment [2]. Currently, microfracture (MF) is a widely applied technique in clinical practice for chondral defects because it is both relatively easy to perform and cost-effective [3]. Although this procedure is beneficial for symptomatic relief and clinical improvement in the short term, its long-term outcome remains unsatisfactory, especially in patients over 50 years old [4]. Notably, chondral defects are typically filled with fibrocartilage derived from blood clots rather than normal hyaline cartilage [5]. One of the possible reasons is that MF alone fails to provide an instructive microenvironmental niche to enhance endogenous stem/progenitor cells (ESPCs) retention and further facilitate chondrogenic differentiation of ESPCs. Hence, there is a dire need to develop a structurally and functionally optimized scaffold that recreates microenvironmental characteristics representative of native cartilage.

3D printing is a promising technology to precisely fabricate customized scaffolds with complex architectures [6]. However, synthetic materials with favorable biocompatibility for 3D printing are relatively limited at present [7]. Common printable materials, such as polylactic acid (PLA), polylactic-co-glycolic acid (PLGA) and poly(ε-caprolactone) (PCL), typically require either high printing temperatures or organic solvents for 3D printing [8]. High-temperature processing will damage the chemical structure of the materials, resulting in reduced mechanical properties. Additionally, the introduction of toxic organic solvents may hamper further biomedical applications due to concerns about solvent residue in the final product [9]. In recent years, waterborne polyurethane (WPU) has attracted great interest from researchers in regenerative medicine because of its desirable properties, such as adjustable chemical structure, tunable mechanical properties, biodegradability, and printability [10]. WPU is a versatile material and its mechanical properties (especially elasticity and flexibility) and degradation rate can be adjusted to match those of newly formed cartilage [9]. Moreover, as water is the dispersion solvent, WPU can be used for water-based 3D printing, which is distinctly advantageous for incorporating bioactive agents into materials to functionalize scaffolds [11]. Hung et al. [12] fabricated a customized waterborne polyurethane scaffold that incorporated the small molecule drug Y27632 and found that the scaffold promoted cell aggregation and cartilage repair. Although there have been some successful reports on WPU in regenerative medicine [13,14], it should be pointed out that WPU scaffolds still inadequately recreate an instructive microenvironment for cell growth and tissue development, owing to minimal cell recognition sites and limited cell-scaffold interactions. Hence, incorporating bioactive materials into WPU could be a good solution to further optimize the biocompatibility of WPU scaffolds. Decellularized ECM is considered an ideal biomaterial since it preserves cartilage-specific matrix compositions that modulate cell behavior [15]. Previous studies [16,17] have shown that decellularized ECM is capable of providing an optimized microenvironment conducive to cell growth. Our previous studies [18,19] also demonstrated the beneficial effects of decellularized ECM on tissue regeneration by providing an instructive microenvironment. Thus, we hypothesized that WPU incorporated with decellularized ECM might compositionally and functionally mimic the cartilage microenvironment and enhance cell-scaffold interactions.

An ideal scaffold should possess a proper pore size and high porosity, both of which have a direct impact on cell behavior [20]. Recently, a hierarchical porous scaffold was reported to be beneficial for nutrient exchange and cell ingrowth and to further promote rapid bone ingrowth and repair [21]. However, it is difficult to fabricate scaffolds with hierarchical macro-microporous structures by conventional 3D printing technologies such as fused deposition modeling (FDM) [22,23]. Fortunately, low-temperature deposition manufacturing (LDM), a new and robust technology to fabricate scaffolds by combining rapid deposition manufacturing and phase separation techniques, could achieve this optimized structure. Detailedly, the polymer solution from the print nozzle can immediately solidify on a cryogenic platform (−20 °C ~ −30 °C), and then the frozen scaffolds are lyophilized to form interconnected microporous structures in the printed fibers [23]. Compared to traditional rapid prototyping technologies, water-based LDM technology has several advantages [10,22,23]. (1) Water-based LDM processes can avoid using toxic organic solvents or toxic photoinitiators for fabrication of the scaffolds. (2) Bioactive agents are conveniently incorporated in scaffolds during the fabrication process. The low-temperature process can well preserve the bioactivities of bioactive agents or materials, which may be denatured in a heating or organic solvent process. (3) In addition to the macroporous structure, the scaffolds fabricated by LDM possess interconnected micropores in the printed fibers. Hierarchical macro-microporous scaffolds can not only allow cell ingrowth and nutrient diffusion but also provide topological cues for cell attachment and adsorption sites for bioactive molecules.

To develop a structurally and functionally optimized scaffold for cartilage repair and regeneration, several strategies were introduced in the current study. (1) WPU was incorporated with ECM to balance the biomechanical properties and biocompatibility of the scaffold. The ECM recreated a conducive microenvironment by providing cartilage-specific biomechanical and biochemical cues that guide cell behavior. Meanwhile, WPU improved the biomechanical properties of the scaffold and slowed its degradation rate. (2) Water-based LDM was adopted to produce precise shapes and structures. This process can prevent the denaturing of the bioactive molecules of the ECM. (3) Lyophilization was used to create a hierarchical macro-microporous structure. Typically, standard 3D-printed scaffolds only possess microporous structures, whereas hierarchical macro-microporous scaffolds fabricated by LDM can further facilitate mass-transport, cell migration and cell attachment.

**Fig. 1:**
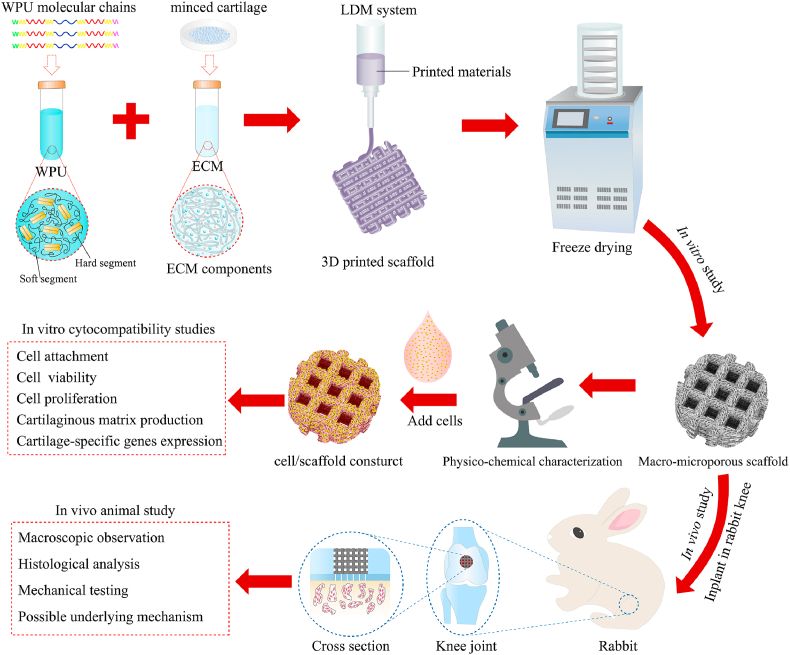
Schematic diagram of the overall study design.

In this study, we utilized WPU and ECM to construct a WPU-ECM hybrid scaffold with a hierarchical macro‐microporous structure by water-based LDM. Then, we examined the physiochemical properties of the scaffolds and their effects on cell behavior and function *in vitro*. Finally, we used WPU-ECM scaffolds combined with MF to repair articular cartilage defects in a rabbit model. Also, we evaluated the regeneration potential of the hierarchical macro‐microporous WPU-ECM scaffold and discussed its clinical applicability in the future. We hope our work provides clinicians and scientists with a new alternative treatment for cartilage defects. An overview of the study design is shown in Fig. 1.

## 2 Materials and methods

### 2.1 Scaffold preparation and fabrication

#### 2.1.1 Preparation of the WPU and ECM

WPU was synthesized by the self-emulsifying method [9]. Prepolymerization was performed using polyethylene glycol (PEG, Mw = 2000 Sinopharm Group, Shanghai, China), polybutylene glycol adipate (PBGA, Mw = 2000, Beijing Organic Chemical Plant, Beijing, China), and isophorone diisocyanate (IPDI, Aladdin, Shanghai, China) at 80 °C under nitrogen for 3 h. Dimethylolpropionic acid (DMPA), as the hydrophilic chain extender, was then added to react for 1 h. Trimethylamine (TEA) was used to neutralize the carboxyl group of DMPA. A deionized aqueous solution of l-arginine was added into the neutralized prepolymer solution under vigorous stirring to obtain WPU dispersion.

The decellularized ECM was prepared by a previously described method with a slight modification [17]. Briefly, articular cartilage was collected from porcine femoral condyles. After ten cycles of freezing (at −80 °C) and thawing (at room temperature), the minced cartilage was homogenized and treated with 0.25% trypsin-EDTA for 24 h. The slurry was washed with phosphate buffer solution (PBS, pH = 7.4) following treatment with nuclease solution containing 50 U/mL DNase and 1 U/mL RNase. Finally, the ECM was washed with deionized water for 3 days to remove the residual reagents.

WPU-ECM hybrid materials were prepared by the dropwise addition of ECM solution into the WPU dispersion at a ratio of 1:1 (v/v) with stirring.

#### 2.1.2 Particle size distribution

The particle size distribution was analyzed by dynamic light scattering (DelsaTM Nano C Particle Analyzer) at 25 °C. WPU and ECM were diluted with deionized water to a concentration of 0.5% prior to measurements.

#### 2.1.3 Fourier transform infrared (FTIR) spectroscopy

A Bruker Tensor 27 spectrometer (Ettlingen, Germany) was used to identify the functional groups in WPU and WPU-ECM. Samples were cut to a size of 1 cm × 1 cm and used for testing in reflection mode. FTIR spectra were recorded in the range of 4000–1000 cm^−1^ with a resolution of 1 cm^−1^.

#### 2.1.4 Rheological characterization

Rheological properties were determined by an Anton Paar MCR302 rheometer (Austria) with a 25 mm diameter plate at 25 °C. The shear viscosity was measured by increasing the shear rate from 0.01 to 1000 s^−1^ at 25 °C. The frequency-dependent storage (G′) and loss (G″) moduli were examined in the frequency range of 0.1–100 Hz.

#### 2.1.5 3D printing and scaffold fabrication

The scaffolds were successfully 3D-printed by LDM. The materials (WPU and WPU-ECM) were transferred into the syringe of the printer and maintained at approximately 4 °C. The temperature of the bottom plate was set at −25~−30 °C. After printing, the frozen scaffolds were lyophilized for 24 h to form the hierarchical macro‐microporous scaffold.

### 2.2 Characterization of 3D-printed scaffolds

#### 2.2.1 Scanning electron microscopy (SEM)

The microarchitectures of the 3D-printed scaffolds were analyzed by SEM (Hitachi S-4800, Tokyo, Japan). The lyophilized scaffolds were mounted on an aluminum stage and sputtered with gold. The microscopic characteristics of the scaffold were observed, and the average macropore size (n = 5) was further analyzed by ImageJ software.

#### 2.2.2 Porosity measurement

The porosities of the scaffolds were determined using the ethanol displacement method [20]. Briefly, the initial volume of ethanol in a cylinder was recorded as V_1_. Then, the scaffold was immersed in ethanol for 10 min until the solution was free of bubbles, at which time the ethanol completely filled the pores, and the new volume was recorded as V_2_. The remaining ethanol volume was recorded as V_3_ when the ethanol -impregnated scaffolds were removed. The porosity was calculated using the following equation: porosity = (V_1_ – V_3_)/(V_2_ – V_3_) × 100%. Five parallel replicates were used for each group.

#### 2.2.3 Water contact angle

The surface contact angle of deionized water was measured on a drop-shaped analysis system (Nuona SL-200B, Shanghai, China) at room temperature. Briefly, deionized water was dropped onto the samples, and images of the drops that settled onto the samples were captured. The shapes of the drops were analyzed to calculate the contact angles. These tests were performed in triplicate.

#### 2.2.4 *In vitro* swelling properties

The lyophilized scaffolds were weighed and immersed in PBS at 37 °C under static conditions. At predetermined time intervals, specimens were withdrawn, and the surface liquid was removed using filter paper. The weights of the scaffolds were noted. The swelling ratio and water uptake were calculated by the following formulas. Five parallel replicates were used for each group.

(1) Swelling ratio = (W_s_ −W_d_) / W_d_; (2) Water uptake (%) = ((W_s_ −W_d_) / W_s_) × 100%

W_d_: Dry weight; W_s_: swelled weight

#### 2.2.5 Degradation *in vitro*

The scaffolds (n = 5) were incubated in PBS with shaking at 37 °C to evaluate their degradation *in vitro*. The mass ratio of the scaffold to the solution was 1:20 and the PBS was changed every two days. The scaffolds were removed after a predetermined amount of time, rinsed with deionized water three times and dried at 60 °C for 12 h to calculate the weight loss ratio. Additionally, the degradation solution was collected for pH measurement.

### 2.3 *In vitro* cytocompatibility studies

#### 2.3.1 Adipose-derived stem cells (ADSCs) isolation, culture and identification

ADSCs were isolated and identified according to previously described methods [24]. Briefly, inguinal adipose tissue from Sprague-Dawley (SD) rats was minced into pieces and digested with 1 mg/mL collagenase (Sigma-Aldrich) for 40 min at 37 °C. Digestion was terminated with expansion medium containing 10% fetal bovine serum (FBS, Corning). ADSCs isolation was centrifuged at 500 g for 10 min, cultured in α-modified minimal essential medium (α-MEM) containing 10% (v/v) FBS and 1% (v/v) penicillin -streptomycin (GIBCO, Biosciences, Ireland) and incubated at 37 °C with 5% CO_2_. ADSCs expanded to passage 3 were used for subsequent experiments. ADSCs were identified using flow cytometry (BD, FACSCelesta™). The antibodies for positive surface markers included CD 29-FITC (BioLegend, 102205) and CD 90-PE (BioLegend, 202523), while the negative markers included CD 34-PE (Biorbyt, orb491006) and CD 45-PE (BioLegend, 202207).

#### 2.3.2 Cell attachment and spreading

ADSCs (a total of 10^6^ cells) were seeded onto the scaffold (10 × 10 × 1 mm^3^) and incubated at 37 °C with 5% CO_2_. After 72 h of incubation, the cell/scaffold constructs were rinsed with PBS three times and fixed with 4% paraformaldehyde for 30 min at room temperature, followed by treatment with 0.3% Triton X-100 solution for 10 min. Phalloidin-rhodamine and DAPI were used to stain the cytoskeletal protein F-actin and nucleus, respectively. Cell morphology was observed using a Leica TCS-SP8 confocal microscope.

#### 2.3.3 Cell viability staining

The viability of the cells on the scaffolds was evaluated by the live/dead assay. Cell/scaffold constructs cultured for 7 days were incubated in sterile PBS containing 2 mM calcein AM and 4 mM ethidium homodimer-1 for 40 min at 37 °C. The distribution and viability of cells were observed under a confocal laser scanning microscope (Leica) with excitation wavelengths of 488 and 568 nm. Live cells were stained fluorescent green, whereas dead cells were stained fluorescent red. Cell viability was calculated according to the following equation: Cell viability = (live cells/total cells) × 100%. The total cell number per region of interest (ROI) was determined by ImageJ software. Three parallel replicates were used for each group.

#### 2.3.4 Cell proliferation assessment

Cell proliferation was evaluated by cell counting kit-8 (CCK-8) assay. Cell suspensions containing 2 × 10^4^ cells were seeded onto the scaffolds and allowed to penetrate into the scaffold. After 4 h of incubation to allow for cell attachment, cell/scaffold constructs were transferred to new 24-well plates and cultured in medium for an additional 1, 4 or 7 days. At predetermined time intervals, a working solution composed of culture medium and CCK-8 reagent (9:1) was added to each well followed by incubation at 37 °C for 1 h. The optical density (OD) values of the working solution (n = 5) were measured at 450 nm using a microplate reader (Beckman, Fullerton, CA).

#### 2.3.5 Biochemical assays for the contents of DNA, GAG and collagen

Cell suspensions (50 μl) were seeded onto the scaffolds at a density of 1 × 10^6^ cells per scaffold, and the constructs were then cultured for 21 days in chondrogenic medium (containing dexamethasone, ascorbate, insulin–transferrin–selenium premix, sodium pyruvate, transforming growth factor β_3_, l-proline, penicillin, and streptomycin). The cell/scaffold constructs on days 0 and 21 were digested with papain buffer for 16 h at 65 °C prior to biochemical assays to determine the contents of DNA, GAG and collagen. DNA content was determined using a PicoGreen DNA kit (Invitrogen, Carlsbad, CA, USA) according to the manufacturers’ instructions. The 1,9-dimethylmethylene blue (DMMB) assay (Genmed Scientific Inc., Shanghai, China) was used to quantify GAG content. Collagen content was determined using a hydroxyproline (HYP) assay kit (Nanjing Jiancheng, Jiangsu, China). These tests were performed in triplicate.

#### 2.3.6 *In vitro* chondrogenic differentiation of ADSCs

Chondrogenic lineage gene expression of ADSCs was analyzed by quantitative real time (RT)-PCR. ADSCs were seeded on tissue culture plates (TCPs), WPU scaffolds and WPU-ECM scaffolds and cultured in serum-free chondrogenic medium for 21 days. ADSCs cultured on TCP served as the control group. Total RNA from ADSCs was isolated using a standard TRIzol procedure. cDNA was synthesized using a PrimeScriptTM RT reagent kit (TaKaRa, RR047A). Quantitative RT-PCR was performed to determine the expression of cartilage-specific genes (Col 2A1, Col 1A1, Sox 9, ACAN) using TB Green® Premix Ex TaqTM II (TaKaRa, RR820A). The relative gene expression was quantified by normalizing gene expression to that of the housekeeping gene GAPDH using the ΔΔCt method. The specific primers for the target genes are shown in Table 1. Three parallel replicates were used for each group.

**Table 1:** Primer sequences of target genes used for RT-PCR.

Target genes	Forward primer 5' → 3′	Reverse primer 5' → 3′	Category
GAPDH	GGCTGCCTTCTCTTGTGACA	TTGAACTTGCCGTGGGTAGA	Housekeeping gene
ACAN	GAAATCCAGAACCTTCGCTCC	AAGTCCAGTGTGTAGCGTGT	Cartilage -related matrix gene
Col 1A1	ATTCACCTACAGCACGCTTG	GATGGAGGGAGTTTACACGAAG	Dedifferentiation marker gene
Col 2A1	TCACGCCTTCCCATTGTTGA	TCAGGTCAGCCATTCAGTGC	Cartilage -related matrix gene
Sox 9	GTTTGACCAATACCTGCCGC	GCCTGTTGCTTTGACATCCA	Transcription factor gene

### 2.4 *In vivo* animal studies

#### 2.4.1 Surgical procedure

The animal study was approved by the local Animal Care Committee of our institute (201903–31). A total of 30 skeletally mature New Zealand White rabbits (male, 3.5–4.0 kg, 8 months old, n = 10 each group) were used as animal models. After general anesthesia and routine preparation, full-thickness cartilage defects (4 mm in diameter, 1.5 mm in depth) were created in the trochlear groove of the distal femur. The microfracture (MF) procedure was performed using a surgical drill to penetrate the subchondral bone in the defects. Rabbits undergoing MF with no scaffold implant were set as the control group. In the experimental groups, the scaffolds (WPU and WPU-ECM) were implanted and press-fitted into the defects. After hemostasis, the joint wound was closed with sutures in layers, and the skin incision was disinfected with iodophor. Postoperatively, analgesia and antibiotics were administered routinely for 5 days. All rabbits were allowed to move freely after surgery. All rabbits were euthanized and evaluated at 3 or 6 months postimplantation.

#### 2.4.2 Macroscopic observations

Macroscopic observations were performed to evaluate defect filling, surface smoothness, and tissue integration by two independent observers blinded to the groupings. The repaired tissues were scored according to the International Cartilage Repair Society (ICRS) scoring system [25] for the macroscopic evaluation of cartilage (n = 5 for each time point).

#### 2.4.3 Histological and immunohistochemical (IHC) analyses

Samples were fixed in 4% paraformaldehyde for 48 h, decalcified with 10% ethylenediaminetetraacetic acid (EDTA) for 7 weeks, embedded in paraffin and cut into 7-μm-thick slices. Hematoxylin and eosin (H&E) staining was employed to assess neotissue morphology and arrangement, and toluidine blue staining was used to identify GAG. Type II collagen (antibody 1:100, Cat# NB600-844, Novus) deposition in the regenerated cartilage was evaluated by standard IHC staining. Histological assessment of the regenerated tissue (n = 3 for each time point) was conducted according to the ICRS histological scoring system [26]. The relative density (integrated optical density IOD/area) was measured to semi-quantify the deposition of type II collagen with the aid of Image-Pro Plus software (n = 3).

#### 2.4.4 Mechanical analysis

The biomechanical performance of the repaired tissues was assessed by indentation tests at room temperature. Samples (n = 3 for each time point) from the central region of the repaired tissues and the native cartilage were isolated and hydrated with PBS. Indentation tests were performed with a dedicated apparatus (ElectroForce 3320; Bose, USA). Young's modulus was calculated according to the slope of the linear fit of the strain-stress curves.

### 2.5 Statistical analysis

All statistical analyses were performed using SPSS v. 25.0 (IBM Corporation; Armonk, New York, USA). For normally distributed data, quantitative data were subjected to one-way analysis of variance (ANOVA) or Student's *t*-test, whereas nonparametric Kruskal-Wallis tests were used for skewed data. Statistical significance was set at a two-sided p-value of <0.05.

## 3 Results

### 3.1 WPU and ECM characterization

#### 3.1.1 Particle size distribution

**Fig. 2:**
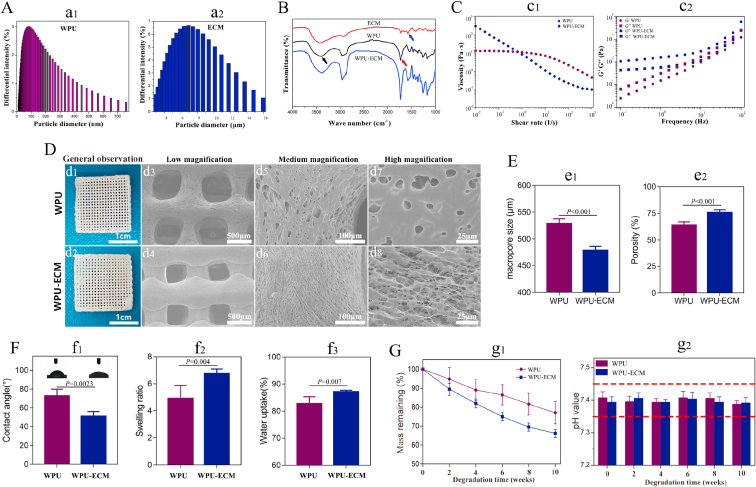
(A) The particle size distributions of WPU and ECM; (B) FTIR spectra of WPU, ECM and WPU-ECM; (C) rheological data for the WPU and WPU-ECM materials; (D) macroscopic and SEM images; (E) macropore sizes and porosities (n = 5); (F) contact angles (n = 3), swelling ratios and water uptake (n = 5); and (G) degradation performance and pH value after degradation (n = 5) in PBS of WPU and WPU-ECM.

The particle size distributions of the WPU and ECM materials are shown in Fig. 2A. The mean particle sizes of the WPU and ECM were 87.5 nm (range 13.5–760 nm) and 6.9 μm (range 3.0–16.0 μm), respectively. Particle size is a prerequisite for 3D printing. These particles were far smaller than the diameter of the print nozzle (traditionally ranging from 100 to 400 μm), indicating that these materials could meet the requirements for printing scaffolds.

#### 3.1.2 FTIR spectroscopy

FTIR spectroscopy was used to characterize the structural changes in the materials. The FTIR spectra of the WPU, ECM and WPU-ECM are shown in Fig. 2B. In the spectrum of WPU, there was a characteristic peak of the imino group (N–H stretching vibration of an aliphatic secondary amine) at 3348 cm^−1^ (black arrow) and a characteristic peak from the carbonyl group (C=O) at 1732 cm^−1^ (red arrow). The peak at 1580 cm^−1^ (N–H bending vibration and C–N stretching vibration of an aliphatic secondary amine, blue arrow) was recognized as a characteristic peak of the amine group in the ECM. The peak at 1580 cm^−1^ increased in intensity with the addition of ECM, demonstrating the successful mixing of WPU and ECM.

#### 3.1.3 Rheological characterization

Shear rate-dependent viscosity was the most direct correlation with the printing process. Both printing materials exhibited a shear-thinning behavior (Fig. 2c_1_), which was considered helpful in allowing continuous material flow though the needle. Of note, the viscosity of WPU-ECM was higher than that of the WPU group with increasing shear rate, which indicated a nonnegligible effect of the extrusion rate on viscosity. WPU-ECM displayed a higher storage modulus (G′) than viscous modulus (G′) at low frequency, while the WPU group revealed the opposite result (Fig. 2c_2_), indicating that the WPU-ECM hybrid material possessed enhanced ability to hold themselves in the nozzle when printing was not in process.

### 3.2 Scaffold characterizations

#### 3.2.1 Microstructures of the scaffolds

The microstructures of the scaffolds were characterized by SEM, as shown in Fig. 2D. Hierarchical macro‐microporous structures were formed in the WPU and WPU-ECM scaffolds. Both scaffolds had uniform macropores, and numerous interconnected microporous structures were observed on the surface of the printed fibers. Yet, the WPU-ECM scaffold showed more highly interconnected micropores than the WPU scaffold. The average macropore size of the scaffold was shown in Fig. 2e_1_ and the micropore size ranged from 5 to 30 μm for both scaffolds. The porosity of the WPU scaffold was 63.7%, while the porosity increased to 75.6% after blending with ECM (Fig. 2e_2_).

#### 3.2.2 Contact angle, swelling ratio and water uptake

The WPU and WPU-ECM scaffolds had contact angles of 72.7 ± 7.11° and 50.95 ± 5.15° (Fig. 2f_1_), respectively. The WPU and WPU-ECM scaffolds yielded swell ratios of 4.9 ± 0.97 and 6.7 ± 0.34 (Fig. 2f_2_) and water uptake values of 82.7 ± 2.62% and 87.1 ± 0.59% (Fig. 2f_3_), respectively, which was in line with the results of the contact angle test. These results indicated that adding ECM into WPU improved the hydrophilia of the scaffold.

#### 3.2.3 *In vitro* degradation

*In vitro* degradation was performed for 10 weeks to evaluate the overall decomposition of the scaffolds (n = 5) over time. The gradual weight loss of each sample was observed with increasing degradation time. After 10 weeks of incubation, the remaining mass ratios of WPU and WPU-ECM were 77.08 ± 5.87% and 66.15 ± 2.17%, respectively (Fig. 2g_1_). It should be noted, however, that degradation would be accelerated *in vivo* due to the presence of several enzymes that would be involved in degradation *in vivo*. Additionally, the pH value of the degradation solution in the WPU and WPU-ECM groups was maintained in the physiological range of 7.35–7.45 (Fig. 2g_2_), which is essential for cell growth and tissue development.

### 3.3 *In vitro* cytocompatibility studies

#### 3.3.1 ADSCs identification

**Fig. 3:**
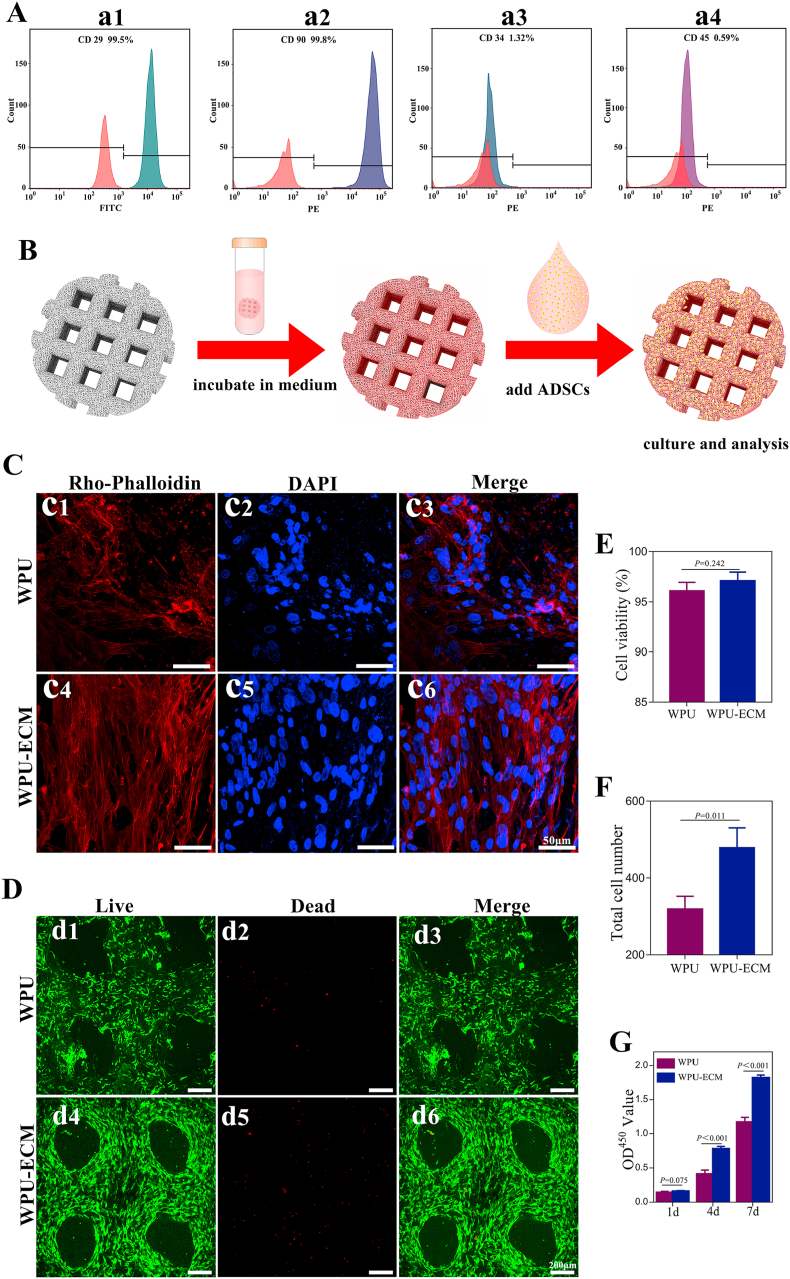
(A) Flow cytometric analysis of MSC-specific surface markers for CD 29, CD 90, CD 34 and CD 45; (B) schematic diagram of ADSCs seeding and culture; (C) confocal morphology (red: F-actin, blue: nucleus); (D) live/dead staining (green: live cells, red: dead cells) of ADSC on the WPU and WPU-ECM scaffolds; (E) cell viability analysis (n = 3) and (F) total cell counting (n = 3) for ADSCs on the different scaffolds based on live/dead cell staining images; and (G) CCK-8 assay (n = 5) of ADSCs after 1, 4 and 7 days of culture.

ADSCs at passage 3 were used to identify the phenotypic characterization. Flow cytometry analysis (Fig. 3A) revealed that ADSCs expressed the cell surface markers CD29 (99.5%) and CD90 (99.8%) but did not express CD34 (1.32%) or CD45 (0.59%), suggesting that these ADSCs were a pure mesenchymal stem cell (MSC) population.

#### 3.3.2 Cell attachment and spreading

To evaluate the cytocompatibility, the scaffolds were incubated with DMEM/F12 for 24 h and then seeded with ADSCs (Fig. 3B). The cytoskeletal protein F-actin was stained with rhodamine-phalloidin to evaluate the cell morphology and cell attachment after 72 h of culture. Compared to the cells on the WPU scaffold, ADSCs on the WPU-ECM scaffold showed a more characteristic fusiform morphology and a significant cell cluster (Fig. 3C), indicating better spreading morphology and cell attachment.

#### 3.3.3 Cell viability

Live/dead staining was used to assess cell viability. After 7 days of culture, the ADSCs grew uniformly on both the WPU and WPU-ECM scaffolds and were arranged along the printed fibers (Fig. 3D). A majority of the cells were stained fluorescent green (live cells), and only very few cells were stained fluorescent red (dead cells). Further quantitative analysis showed that both scaffolds exhibited a cell viability greater than 95%, indicating that both scaffolds had good cytocompatibility and were suitable for cell growth. By counting the total number of cells on the scaffolds, we found that the total cell number on the WPU-ECM scaffold (479 ± 30) was significantly higher than that on the WPU scaffold (319 ± 19).

#### 3.3.4 Cell proliferation

The proliferation of ADSCs on the WPU and WPU-ECM scaffolds was further quantitatively measured by CCK-8 assay after 1, 4 and 7 days of culture. The absorbance values in both groups increased with incubation time, and the absorbance values on the WPU-ECM group were greater than those on the WPU group after 4 and 7 days of incubation. These results were consistent with the cell counts from live/dead staining, demonstrating that the cell proliferation ability on the WPU-ECM scaffold was significantly higher than that on the WPU scaffold.

### 3.4 *In vitro* chondrogenic differentiation of ADSCs on the scaffolds

**Fig. 4:**
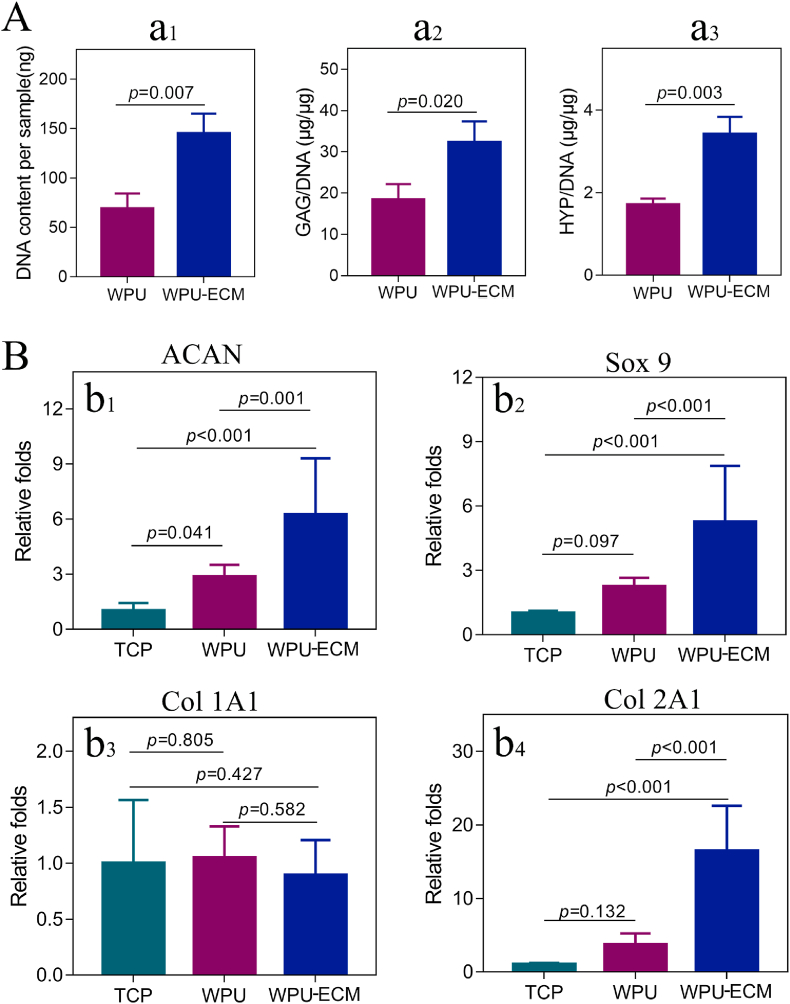
(A) DNA content, GAG/DNA, and HYP/DNA of the cell/scaffold constructs after 21 days of incubation (n = 3); and (B) expression of ACAN, Sox 9, Col 1A1 and Col 2A1 of the ADSCs on WPU and WPU-ECM scaffolds compared to ADSCs on the tissue culture plate (TCP) (n = 3).

The capacity of the scaffolds to promote *in vitro* chondrogenic differentiation of ADSCs was evaluated by cartilaginous matrix production and the expression of hyaline cartilage-specific genes. Collagen and GAG are the main components of the ECM of cartilage, so both were assessed to evaluate cartilaginous matrix production by ADSCs on the scaffolds. DNA quantification (Fig. 4a_1_) revealed that the DNA content was significantly higher in the WPU-ECM scaffold than in the WPU scaffold, which further supported the cell proliferation results. After incubation in chondrogenic medium for 21 days, total collagen (HYP) and GAG were quantified and normalized to the DNA content. The WPU-ECM scaffold showed significantly higher levels of collagen and GAG deposition than the WPU scaffold (Fig. 4a_2_ and 4a_3_). The matrix production results were further supported by the gene expression of chondrogenic markers. As shown in Fig. 4B, the gene expression levels of Col 2A1, SOX 9, and ACAN were observed to be higher in the WPU-ECM scaffolds after 21 days of culture than in the WPU group. No significant difference was found in COL 1A1 expression between the WPU and WPU-ECM scaffolds. Limited to measurement methods, we could assess only the total collagen production rather than differentiating between type I and type II collagen production. However, the RT-PCR results provided evidence that the expression of type I collagen was lower than that of type II collagen, suggesting that type II collagen accounted for a large proportion of the total collagen. Taken together, these results indicated that WPU-ECM could provide a favorable microenvironment for chondrogenic differentiation of ADSCs.

### 3.5 *In vivo* animal study

#### 3.5.1 Gross morphological and histological evaluation

**Fig. 5:**
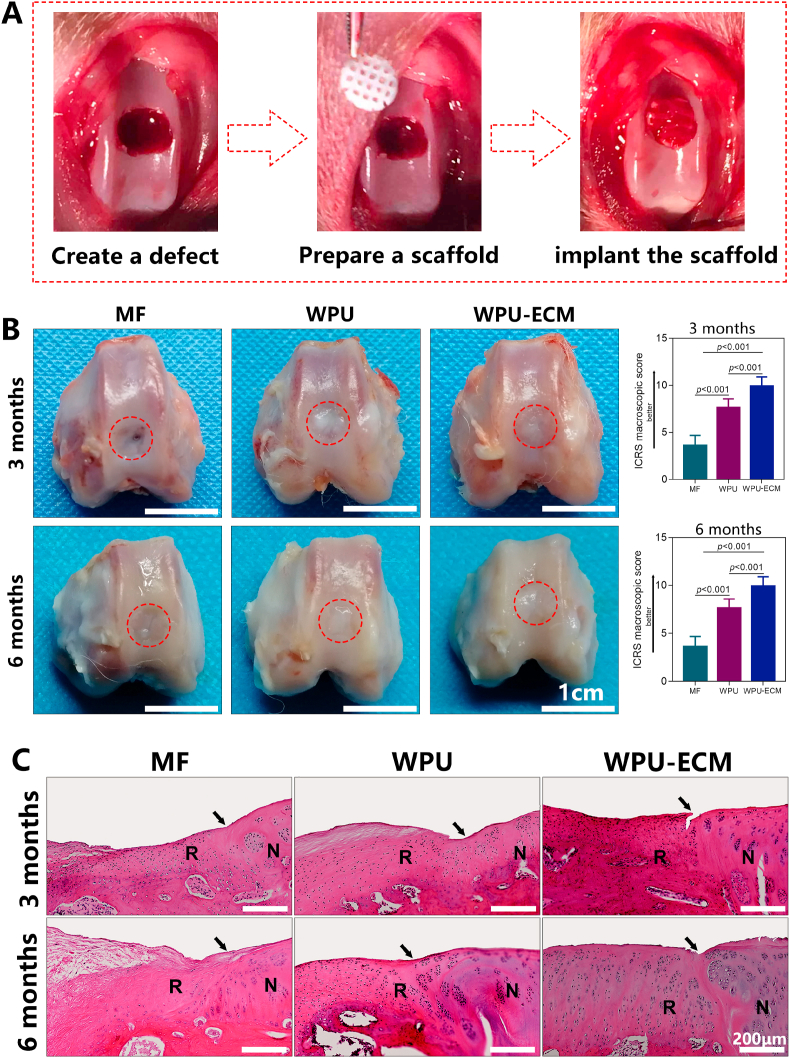
(A) Cartilage defect creation and scaffold implantation; (B) macroscopic observations and ICRS macroscopic scores of the regenerated cartilage at 3 and 6 months after implantation (n = 5 for each time point); and (C) H&E staining images of the repaired cartilage at 3 and 6 months postsurgery. (R: repaired cartilage; N: normal cartilage. The arrows indicate the margins between the normal cartilage and the repaired cartilage.)

To determine the role of the cell-free scaffolds in cartilage repair in a preclinical manner, we implanted the scaffolds into cartilage defects in a rabbit model and evaluated the outcome at 3 and 6 months after implantation (Fig. 5A). By 1 week after surgery, all of the animals resumed locomotion and weight bearing. None of the animals died from anesthesia, surgical trauma or postoperative complications. The synovial fluid was clear, and no intraarticular inflammation, infections or swelling were found in any of the groups.

As shown in Fig. 5B and C, there was little regeneration within the defect in the MF control group at 3 months after surgery. Although the defects were partially filled at 6 months, the neotissue was loose fibrotic tissue with a disordered structure. The WPU and WPU-ECM scaffolds were almost completely degraded 3 months after implantation. Generally, the WPU group showed gradual improvement in the repaired tissues in the defect region over time. However, the repaired tissue was uneven, and the boundary was still distinct. Additionally, the neotissue was thinner than the height of the surrounding cartilage, and cell arrangement appeared in a clustered manner. In contrast, in the WPU-ECM group, the defect was almost entirely filled with neocartilage tissue, showing a smoother surface and better integration with the surrounding host cartilage than in the WPU group. In particular, the repaired tissue showed a healthy appearance with columnar cell distribution and a glossy white color after 6 months, which was similar to native cartilage.

In accordance with the gross observations, the ICRS macroscopic evaluation score showed that the repaired cartilage was markedly better at 6 months compared with that at 3 months in all of the scaffold groups, and the score in the WPU-ECM group was higher than those in the WPU and MF groups at 3 and 6 months postsurgery.

#### 3.5.2 Cartilage-specific staining

**Fig. 6:**
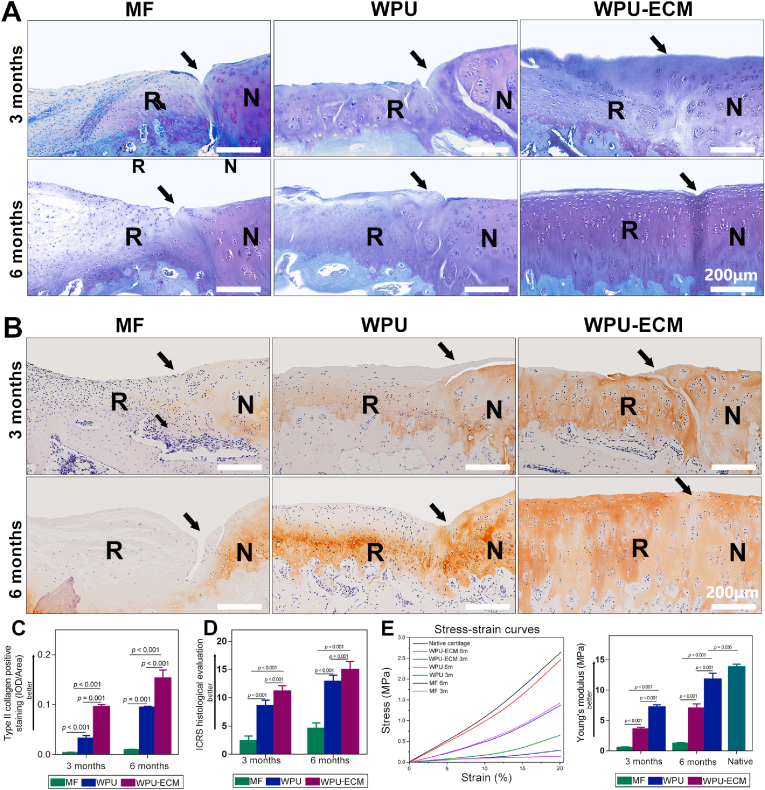
(A) Toluidine blue staining; (B) IHC staining for type II collagen (R: repaired cartilage; N: normal cartilage. The arrows indicate the margins between the normal cartilage and the repaired cartilage.); (C) Values for integrated optical density (IOD) per area of type II collagen at 3 and 6 months postsurgery (n = 3). (D) ICRS histological score (n = 3 for each time point) and (E) biomechanical assessment (n = 3 for each time point) of the repaired cartilage at 3 and 6 months postimplantation.

The cartilage-specific matrix was identified by toluidine blue (Fig. 6A) and type II collagen IHC staining (Fig. 6B). Toluidine blue did not stain the neotissue in the MF group. Light toluidine blue staining was observed in the WPU group at 3 months after implantation and the staining intensity increased after 6 months. In the WPU-ECM group, toluidine blue staining was positive at both 3 and 6 months after surgery, and was comparable to the staining of the surrounding native cartilage at 6 months. Similarly, IHC staining for type II collagen was positive in the WPU-ECM group, and the staining intensity was stronger than that in the other two groups at 3 and 6 months after the operation. The IOD/area for type II collagen further supported that type II collagen deposition in the WPU-ECM group was higher than that in the WPU and MF groups at 3 and 6 months postsurgery (Fig. 6C). These results suggested that hierarchical macro‐microporous WPU-ECM scaffolds combined with MF promoted hyaline cartilage repair and regeneration.

Generally, as the repair process progressed, the histological scores gradually improved in all of the scaffold groups, and there were higher histological scores for the repaired tissues in the WPU-ECM group than in both the MF group and WPU group at 3 and 6 months after implantation. Taken together, these results demonstrated that WPU-ECM enhanced hyaline cartilage regeneration *in vivo*.

#### 3.5.3 Biomechanical assessment

Young's modulus was measured to evaluate the biomechanical properties of the regenerated cartilage (Fig. 6E). Generally, the Young's moduli of the repaired tissue improved over time in all groups. The neocartilage had higher Young's moduli at 6 months than at 3 months in each group. The Young's modulus of the regenerated cartilage in the WPU-ECM group was significantly higher than those of the WPU and MF groups at both time points. Moreover, the Young's modulus of the repaired cartilage was similar to that of native articular cartilage at 6 months after implantation in the WPU-ECM group.

## 4 Discussion

This study used hierarchical macro‐microporous WPU-ECM scaffolds combined with MF to stimulate *in situ* cartilage regeneration. The results demonstrated that (a) LDM can be used to fabricate scaffolds with hierarchical macro‐microporous structures; (b) incorporation with ECM not only increased the interconnected micropores and porosity but also improved the hydrophilia of the scaffold, further positively regulating cell behavior and function; and (c) hierarchical macro‐microporous WPU-ECM scaffolds combined with MF enhanced hyaline cartilage regeneration in rabbits.

In the current study, WPU and ECM were used to construct a novel hybrid scaffold with hierarchical macro‐microporous structures. We found that the addition of ECM could markedly increase both the interconnected micropores and porosity, which could be ascribed to the higher water content of the ECM than that of the WPU. The concentration of the WPU used for printing in this study was 30% (w/v), while the concentration of the final ECM was approximately 3%. Previous studies [20,27] have shown that the porosity and spatial architecture of scaffolds have direct implications on cell proliferation, differentiation, and ECM production. Regarding the preparation methods for hierarchical porous scaffolds, LDM technology is promising and shows an apparent advantage. On the one hand, conventional fabrication methods, such as fiber bonding, solvent casting, and particulate leaching, could realize porous structures, but all of them fail to control the precise and complex architectures that can be achieved by 3D printing techniques [28,29]. On the other hand, most of the conventional 3D-printed scaffolds were designed with uniform macroporous structures rather than hierarchical porous structures. In addition to the controlled macroporous structure, the scaffolds fabricated by LDM possessed interconnected micropores in the printed fibers [23,30]. This hierarchical porous structure was shown to have several advantages over the uniform porous structure [31]. Hierarchical porous scaffolds can not only allow cell ingrowth and nutrient diffusion but also provide topological cues for cell attachment and adsorption sites for bioactive molecules [23].

Adding ECM into WPU also increased the hydrophilicity of the scaffold because the main components of ECM are collagen and GAGs, both of which are superabsorbent materials [32]. The *in vitro* cytocompatibility studies demonstrated that the WPU-ECM scaffold was favorable for cell attachment, spreading and proliferation. Additionally, our findings showed that the WPU-ECM scaffold promoted extracellular matrix production and upregulated the expression of chondrogenic markers. We speculate that these results can be explained as follows. (i) An optimized microstructure and the hydrophilic properties of the WPU-ECM scaffold could construct a suitable 3D environment that is conducive to cell adhesion and proliferation [33,34]. (ii) Decellularized ECM is perceived to be an ideal biomaterial because it can embrace almost all of the features and profiles of natural ECM [15,16]. The incorporation of decellularized cartilage ECM may closely mimic the microenvironment of cartilage. Therefore, ADSCs are activated and modify their behavior and functions in response to biochemical and mechanical cues in the ECM.

In the *in vivo* study, the repaired tissues in the MF group were fibrous tissue with poor integration and a disordered structure, which was consistent with previous studies [35,36]. A possible reason for these observations is that MF alone fails to provide an instructive microenvironmental niche to enhance ESPCs retention, proliferation and chondrogenic differentiation [5]. This finding also emphasized the potency of the scaffolds during cartilage repair. The regenerated cartilage in the WPU-ECM group was superior to that in the WPU group in terms of histological structure and biomechanical properties at 3 and 6 months postimplantation. This finding was in line with the *in vitro* study results, highlighting the significant role of the WPU-ECM scaffold in the process of cartilage regeneration.

Regarding clinical applications, although cell-based tissue-engineered strategies have been shown to be effective in repairing cartilage defects [35,36] and great progress has been made in cell-loaded 3D printing [37,38], cell-free scaffolds have attracted more attention for translation, and the acellular concept has also been verified by scientists [39,40]. On the one hand, one of the foremost concerns in cell therapy is safety [41]. Specifically, cell transplantation may introduce potential risks to hosts. To date, there are no standardized manufacturing guidelines for the isolation, expansion, preservation, and delivery of stem cells [41]. Besides, stem cells from different donors can vary in performance. On the other hand, cell-based products frequently necessitate more comprehensive and longer-term evaluation of cell-scaffold interactions prior to regulatory approval [42]. In contrast, cell-free scaffolds have the advantages of cost-effectiveness and ease of packaging, storing, and shipping [43,44]. The cell-free paradigm can not only avoid several serious concerns about cell transplantation, but also accelerate therapeutic translation [43,45], and thus may be attractive and have great clinical application prospects.

**Fig. 7:**
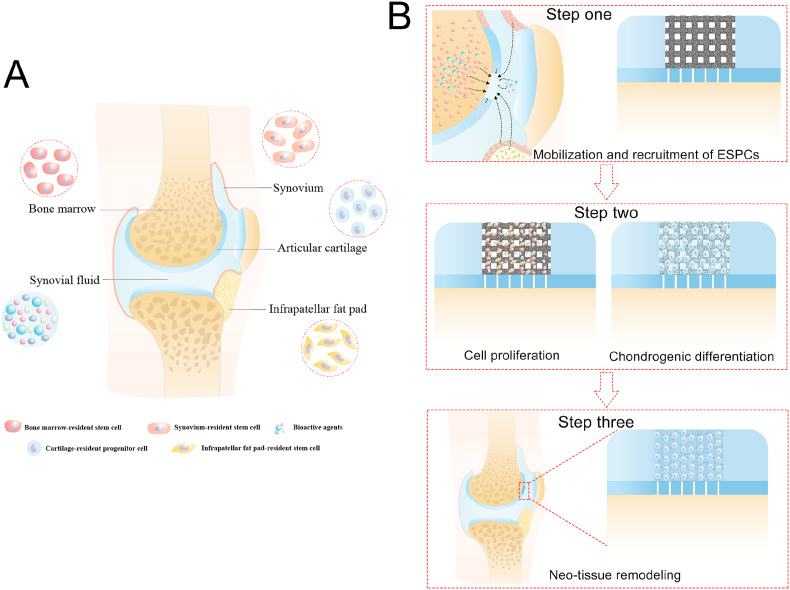
(A) Endogenous stem/progenitor cells (ESPCs) and bioactive agents within the knee joint participated in cartilage repair and regeneration; and (B) proposed schematic of the cartilage repair process that occurs in the hierarchical macro-microporous WPU-ECM scaffold.

Cell-free strategies usually involve recruiting ESPCs to participate in tissue repair/regenerative processes [46]. Previous studies have confirmed the existence of joint-resident ESPCs and their contributions to cartilage regeneration [47]. These joint-resident ESPCs include bone marrow-resident stem cells, synovium-resident stem cells, infrapatellar fat pad-resident stem cells and cartilage-resident progenitor cells (Fig. 7A). There is an inherent repair capacity in the body to recruit ESPCs in response to injury, yet this capacity is comparatively insufficient [48]. A variety of strategies are currently available to facilitate the mobilization and homing of ESPCs, such as chemokines, growth factors, functional peptides, bioactive small molecules, and MF. Among them, MF has the advantages of simplicity, repeatability and cost-effectiveness and is more easily applied in clinical practice [46,49,50]. Therefore, the paradigm of optimized WPU-ECM scaffolds and MF shows great potential for clinical applications in the future.

Based on our results, we proposed a possible mechanism of cartilage regeneration, which can be summarized in three main stages (Fig. 7B). (1) Step one: mobilization and recruitment of ESPCs from their native niche to an injury site. In this study, MF was used to mobilize and recruit endogenous cells. Besides bone marrow-resident stem cells enrichment, microfracture can also facilitate the release of bioactive agents, such as platelet-derived growth factor, bone morphogenetic proteins, and stromal cell-derived factor-1, which can further facilitate other joint-resident ESPCs homing [46]. (2) Step two: ESPCs proliferation and differentiation into chondrocytes in the hierarchical macro‐microporous WPU-ECM scaffold. The WPU-ECM scaffold serves as a micronich that provides biochemical and biomechanical cues to encourage cell attachment, proliferation and chondrogenic differentiation. Additionally, the facilitation effect would be further enhanced by growth factors released from bone marrow and synovial fluid. (3) Step three: neotissue remodeling and maturation. In this stage, an increasing amount of cartilage-specific ECM is produced with prolonged implantation time, and the scaffolds gradually degrade. Cells interact with their environment and convert mechanical stimuli from physiological loading into biochemical activity, resulting in ECM deposition, remodeling, maturation and the further formation of functional tissue.

Although great efforts were made to perform our current research, there were still several limitations that need to be addressed. First, a drawback of this animal model is that rabbits exhibit higher endogenous healing potential than other large animals and humans, making evaluation of the translational potential difficult. Longer-term outcomes of this scaffold in a large animal model (sheep) will be examined in our next experiment. Second, the ECM was made from decellularized porcine articular cartilage owing to its low cost and ease of availability. Allogenic decellularized ECM could be readily applied in clinical practice. Third, *in vivo* degradation of the scaffold was not evaluated in our work. Despite these limitations, this study still demonstrates that the hierarchical macro‐microporous WPU-ECM scaffold combined with MF can successfully promote hyaline cartilage regeneration.

## 5 Conclusions

In this study, we successfully fabricated a hierarchical macro-microporous WPU-ECM scaffold using LDM. The WPU-ECM scaffold provided a suitable microenvironment for cell attachment, proliferation, and differentiation *in vitro*. Moreover, the cell-free WPU-ECM scaffold combined with MF helps to promote *in situ* cartilage regeneration *in vivo*. These results demonstrated that hierarchical macro-microporous WPU-ECM scaffolds combined with MF may be a promising alternative for cartilage regeneration in the future.

## Authors’ contribution

All authors were involved in drafting the paper, and all authors approved the final version to be published.

## Disclosure statement

All authors declare that there is no conflict of interests regarding the publication of this paper.

## CRediT authorship contribution statement

**Mingxue Chen:** Methodology, Investigation, Writing - original draft, Visualization, Formal analysis, Funding acquisition. **YangYang Li:** Methodology, Investigation, Writing - original draft, Visualization, Formal analysis. **Shuyun Liu:** Resources, Visualization, Formal analysis, Methodology. **Zhaoxuan Feng:** Investigation, Resources, Data curation. **Hao Wang:** Investigation, Data curation, Validation. **Dejin Yang:** Resources, Formal analysis, Funding acquisition. **Weimin Guo:** Methodology, Writing - review & editing, Validation. **Zhiguo Yuan:** Methodology, Writing - review & editing, Software. **Shuang Gao:** Methodology, Writing - review & editing, Software. **Yu Zhang:** Methodology, Writing - review & editing. **Kangkang Zha:** Investigation, Data curation. **Bo Huang:** Investigation, Data curation, Validation. **Fu Wei:** Investigation, Data curation. **Xinyu Sang:** Investigation, Data curation. **Qinyu Tian:** Investigation, Data curation. **Xuan Yang:** Investigation, Software. **Xiang sui:** Investigation, Data curation. **Yixin Zhou:** Conceptualization, Writing - review & editing, Funding acquisition. **Yufeng Zheng:** Conceptualization, Writing - review & editing. **Quanyi Guo:** Conceptualization, Writing - review & editing, Supervision, Project administration, Funding acquisition.

## Declaration of competing interest

We declare that there is no conflict of interests regarding the publication of this paper entitled, “Hierarchical Macro‐microporous WPU-ECM Scaffolds Combined with Microfracture Promote in Situ Articular Cartilage Regeneration in Rabbits”.
